# Wisconsin Dental College Again

**Published:** 1882-07

**Authors:** 


### WISCONSIN DENTAL COLLEGE AGAIN.

The general impression, for the last year or more, that this concern had been so thoroughly ventilated that its power for harm had been destroyed. This may be true, in the main, so far as this country is concerned, yet it seems by the following extract from a private letter, that still an effort is made to push this nefarious business abroad.

The question has been asked several times, "What has the dental profession in Wisconsin been doing for the last two years, that this scandalous institution has not been wiped out?" It is easier to ask such a question, however, than to do the work implied. But to the letter:

GLASGOW, Scotland, May 27, 1882.

MY DEAR DOCTOR:—I trust you will excuse the liberty I have taken in writing about the following: This morning I had a communication from the Wisconsin Dental College, of which Dr. Morrison is president, expressing a desire to confer on me the honorary degree of D.D.S. You will, I have no doubt, remember the conversation we had, when I had the honor of a visit from you, on the subject of my obtaining the honorary degree.

I wish to know if the suggestion came from you, or is the diploma not worth the paper it is printed on?

I should like very much to have a degree, but it must come from a reputable institution, and in an honorable way. An early reply will very much oblige

Yours truly, * * *

The dental journals of Great Britain and the Continent, will do valuable service to the profession and the public by publishing such a statement about this concern as will place it in its true light before those who are interested, or are likely to be entrapped by it.

